# GPR30, but not estrogen receptor-α, is crucial in the treatment of experimental autoimmune encephalomyelitis by oral ethinyl estradiol

**DOI:** 10.1186/1471-2172-11-20

**Published:** 2010-04-19

**Authors:** Melissa A Yates, Yuexin Li, Peter J Chlebeck, Halina Offner

**Affiliations:** 1 Neuroimmunology Research, Portland VA Medical Center, Portland, OR, USA; 2 Department of Neurology, Oregon Health & Science University, Portland, OR, USA; 3 Department of Molecular Microbiology & Immunology, Oregon Health & Science University, Portland, OR, USA; 4 Department of Anesthesiology and Perioperative Medicine, Oregon Health & Science University, Portland, OR, USA

## Abstract

**Background:**

Remission of multiple sclerosis during periods of high ovarian hormone secretion (such as pregnancy) has led to a great deal of interest in the potential for estrogens to treat autoimmune disease. Previous work has established that 17β-estradiol can inhibit onset of experimental autoimmune encephalomyelitis (EAE), while ethinyl estradiol (EE) can reduce the severity of established disease. In the current study, the influence of estrogen receptor-α (ERα) and the G-protein coupled estrogen receptor (GPR30 or GPER) on EE's ability to treat EAE was explored.

**Results:**

EE reduced disease severity in wild-type and ERα knockout (ERKO) mice, but did not alter disease in the GPR30KO group. Production of anti-inflammatory IL-10 increased in EE-ERKO mice (which showed reduced disease) but not in EE-GPR30KO mice (who did not have improved disease).

**Conclusions:**

Differential production of IL-10 following EE treatment in ERKO and GPR30KO animals may be responsible for the distinctly different effects on disease severity. Increased IL-10 in ERKO-EE compared to ERKO-Controls is likely to be an important factor in reducing established disease. The inability of EE to reduce disease in GPR30KO mice indicates an important but still undefined role for GPR30 in regulating immune reactivity.

## Background

Multiple sclerosis (MS) is an inflammatory demyelinating disease, which affects women more often than men [1]. Although women more frequently suffer from MS, remission of disease symptoms often occurs at times when ovarian hormone levels are high, such as during pregnancy [2,3]. Using experimental autoimmune encephalomyelitis (EAE), the animal model of MS, previous work has examined the mechanisms involved in estrogen's effects on the immune system. When given prior to immunization, 17β-estradiol (E2) protects against development of EAE, primarily by decreasing the production of inflammatory cytokines, increasing anti-inflammatory cytokines (including IL-10), and through expansion of regulatory T cells (such as FOXP3+ and PD1+ cell populations) [4-8]. Investigations into the estrogen receptors (ER) involved in the effects of E2 have largely implicated ERα and the G-protein coupled estrogen receptor (GPR30), while E2 can still protect mice lacking ERβ [9,10].

Although E2 can protect against the development of EAE when given subcutaneously prior to immunization with myelin peptide, it cannot treat established disease (Offner, unpublished data). Conversely, ethinyl estradiol (EE) can reduce disease severity when given orally at the onset of disease symptoms [11]. EE is a synthetic estrogen, frequently used in oral contraceptives, which maintains its bioavailability after oral dosing, unlike E2 [12]. In addition to differences in bioavailability, it is possible that differential activation of estrogen receptors may be involved in the treatment ability of EE compared to the ineffectiveness of E2. The protective effects of E2 are lost in mice lacking ERα (ERKO) but maintained in mice lacking ERβ [9]. Although disease onset is delayed in GPR30-deficient (GPR30KO) mice pre-treated with E2, much of the protective effect of E2 on disease severity is lost [10]. In the current study, we used mice lacking ERα and GPR30 to explore their role in the ability of EE to treat EAE. Given the importance of changes in the balance of pro- and anti-inflammatory cytokines and the role of regulatory T cell populations in the action of E2, we will also examine whether the effects of EE rely on similar mechanisms.

## Methods

### Animals

Wild-type (WT) female C57BL/6 mice were obtained from Harlan Laboratories (Houston, TX). ERKO and GPR30KO mice were bred using in-house colonies. The ERKO strain originated with the Korach laboratory and is on a C57BL/6 background. The generation of ERKO [13] and GPR30KO mice (also on a C57BL/6 background) [14] have previously been described. All animals were housed in the Animal Resource Facility at the Portland VA Medical Center and experiments were performed in accordance with institutional guidelines. Animals were ovariectomized at 7-8 weeks of age and immunized 1 week later. Mice were immunized with 200 μg of MOG 35-55 peptide (PolyPeptide laboratories; San Diego, CA) in 400 μg of complete Freund's adjuvant. Mice were also injected with pertussis toxin at the time of immunization (d0; 75 ng) and d2 (200 ng). Animals were observed daily and scored for disease severity according to the following scale: 0 = normal, 1 = limp tail, 2 = mild hindlimb weakness, 3 = moderate hindlimb weakness, 4 = severe weakness or partial paralysis, 5 = complete hindlimb paralysis, 6 = moribund. Experiments were completed twice. The total number of animals in each group was WT: N = 12 per group, ERKO: Control (Ctrl) N = 9, EE N = 10, GPR30KO: Ctrl N = 13, EE N = 12.

### Ethinyl estradiol treatment

At the onset of EAE (first day with a score of 1 or greater, approximately D11-12 post-immunization), animals were randomly assigned to receive either ethinyl estradiol (1 mg/day) or control treatment. Treatments were given in a 100 μl volume of olive oil by oral gavage.

### Splenocyte analyses

Spleens were collected at D26 post-immunization. Tissues were homogenized through fine mesh screens and single cell suspensions were used for cytokine assay and flow cytometry. Splenocytes (4 × 10^6^/ml) were cultured and stimulated with MOG 35-55. Supernatants were collected 48 hrs later and frozen at -80°C until the time of cytokine assay. IL-10 and IL-17 were measured using a bio-plex luminex kit (Bio-Rad; Hercules, CA). For flow cytometry, cells were stained using: CD4-FITC and intracellular staining for FOXP3-APC and PD-1PE was completed after fixation/permeabilization (EBiosciences; San Diego, CA). Cells were then run on a FACSCalibur (Becton Dickson).

### Statistical analysis

The day of onset, clinical disease scores, and cumulative disease index within each genotype (Control vs EE) was compared using *t *test, as well as WT- Ctrl comparisons to Control in each genotype. Evaluation of changes in cytokine levels within each genotype were also completed using *t *tests; the accepted level of significance was p < .05.

## Results

### Disease scores

**Figure 1:**
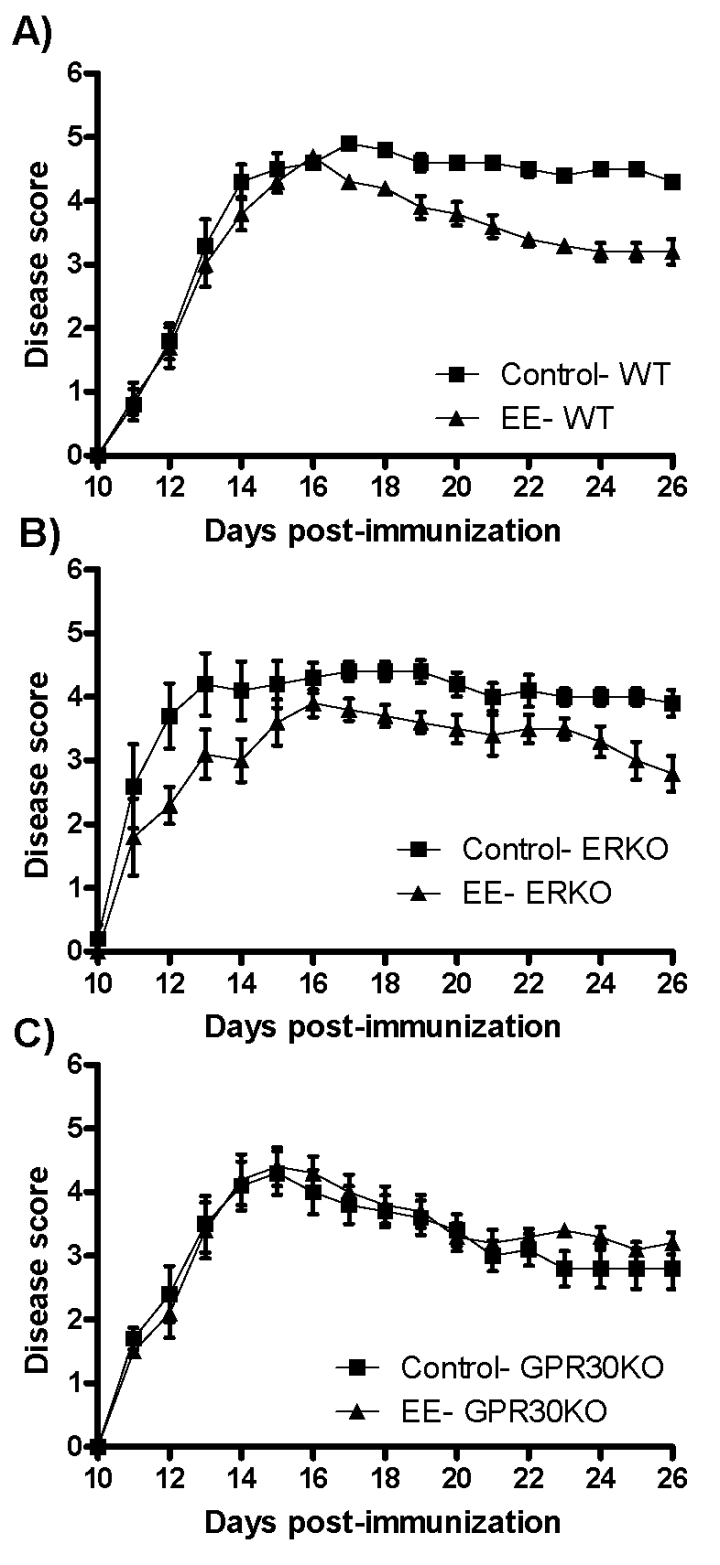
**Treatment with ethinyl estradiol (EE; 1 mg/day) reduced disease severity in ovariectomized wild-type (WT) mice (A) and estrogen receptor-α knockout (ERKO) mice (B), while mice lacking the G-protein coupled estrogen receptor (GPR30KO; C) showed no improvement in disease severity**. WT: N = 12 per group. ERKO: Ctrl N = 9, EE N = 10. GPR30KO: Ctrl N = 13, EE N = 12. * indicates p < .04

From D17-D26, disease was significantly inhibited in WT mice treated with EE (p < .03, Figure 1A). While cumulative disease index (CDI) was also reduced (p < .01), disease onset and peak disease scores did not differ (Table 1). ERKO mice treated with EE also had reduced disease severity (p < .04; D17-20, 23-26, Figure 1B). Peak disease scores and CDI were also reduced in ERKO EE animals (p < .03, Table 1). However, GPR30KO animals showed no alteration in disease course with EE treatment (Figure 1C and Table 1). Within Ctrl mice, there was no difference in peak disease severity or onset between WT-Ctrl and GPR30KO-Ctrl mice. However, GPR30KO mice did show lower average disease scores (D16-26) and a reduced CDI compared to WT (p < .02). There were no differences between WT-Ctrl and ERKO-Ctrl mice.

**Table 1:** Disease was reduced in EE treated WT and ERKO mice compared to their respective controls.

		**CDI**	**Onset**	**Peak**
		
**WT:**	**Ctrl**	64.5 (± 2.5)	12.3 (± 0.4)	5.1 (± 0.1)
		
	**EE**	54.0* (± 1.8)	12.0 (± 0.5)	4.8 (± 0.1)
		
**ERKO:**	**Ctrl**	60.5 (± 4.0)	11.7 (± 0.4)	4.8 (± 0.2)
		
	**EE**	48.5* (± 3.1)	12.2 (± 0.4)	4.2* (± 0.2)
		
**GPR30KO:**	**Ctrl**	47.6 (± 5.3)	11.5 (± 0.4)	4.5 (± 0.3)
		
	**EE**	51.2 (± 4.2)	11.0 (± 0.4)	4.6 (± 0.6)

Cumulative disease index (CDI) was reduced in EE-WT and EE-ERKO mice compared to their respective control groups. Peak disease severity was only reduced in ERKO-EE mice. Onset did not differ between any EE and control groups. * indicates p < .03

### Cytokine analysis and flow cytometry

**Figure 2:**
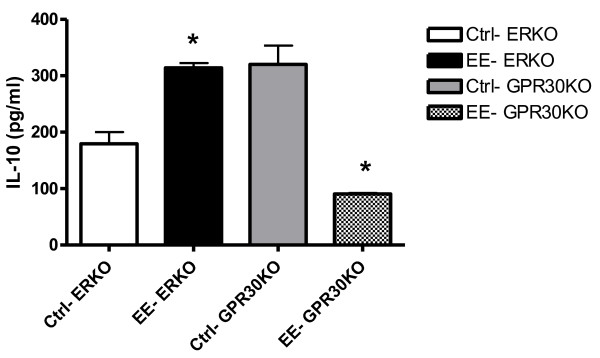
**Cytokine analysis showed increased IL-10 secretion in ERKO-EE mice, but levels decreased in GPR30KO-EE mice compared to Controls**. * indicates p < .03. Results are representative of experiments completed twice.

In the presence of antigen, IL-10 production decreased in EE-GPR30KO mice (p < .02), but increased in IL-10 secretion for EE-ERKO splenocytes (p < .03) compared to their respective controls (Figure 2). IL-17 levels did not change significantly between EE or Control mice in either the ERKO or GPR30KO strains. No significant changes were seen in FOXP3+ or PD1+ cells in any strain (data not shown).

## Discussion

The present study sought to explore the role of estrogen receptors in the ability of EE to reduce EAE disease severity. Expression of ERα and ERβ have been documented on a variety of immune and CNS cells, including T cells, macrophages, microglia, oligodendrocytes, and astrocytes [15-19], which are involved in EAE disease severity. Recent investigation are also beginning to delineate expression of GPR30 in the CNS and immune system, but results remain mixed [20]. The use of mice lacking ERα or GPR30 allowed us to more fully examine estrogen receptor involvement in the pathways affected by EE treatment in EAE.

The effects of EE on WT mice in the current study are in agreement with previous work demonstrating decreased disease severity [11]. Expression of GPR30 seems to be crucial in the ability of EE to reduce disease, as no treatment effect was observed in GPR30KO mice while ERKO mice maintained their ability to respond to EE treatment of EAE. The difference in disease scores between GPR30KO-Ctrl and WT-Ctrl mice is intriguing but the underlying cause remains unclear. While our primary interest in the current experiment was the effect of EE within each genotype, future work will further investigate this divergence between the genotypes.

In an effort to determine specific changes responsible for the disease inhibition in EE-ERKO but not EE-GPR30KO mice, we examined cytokine production by splenocytes in response to antigen, specifically the anti-inflammatory cytokine IL-10 as well as IL-17 (which plays a role in inflammation and pathogenesis in EAE; [21]). Although IL-17 did not change in either strain, secretion of IL-10 was increased in EE-ERKO mice compared to Controls, but decreased in EE-GPR30KO mice. This difference in IL-10 production may be an important contributor to the disease reduction in EE-ERKO mice compared to the GPR30KO mice which did not improve with EE treatment. Increased IL-10 secretion is frequently associated with improvement in disease [22,23], while IL-10 deficient animals have a greater T cell response to antigen and develop more severe EAE compared to WT mice [24]. The subset of cells responsible for this differential secretion of IL-10 in the current experiment is unclear however, and warrants further investigation.

Our data in the WT groups also demonstrates the differential mechanisms of EE action compared to 17β-estradiol, which can prevent but not treat EAE after disease onset. While increases in the FOXP3+ and PD1+ regulatory T cell populations seem to be primary mechanisms of action for 17β-estradiol protection against EAE [6-8], no changes were found in EE treated animals of any strain in the current study. In addition, ERα expression is necessary for 17β-estradiol protection against EAE [9] but is not crucial in EE treatment since EE-ERKO mice still improved with EE treatment in the absence of ERα.

## Conclusions

The lack of disease improvement in EE treated GPR30KO mice indicates a crucial role for GPR30 in altering disease severity, which is likely to be related to the production of IL-10. Further investigation of the mechanisms behind the change in IL-10 production will be necessary to understand the cell populations responsible. In addition, the responsiveness of ERKO mice to EE treatment indicates that ERα is not a major factor in disease inhibition due to EE treatment. The presence of both GPR30 and ERβ in ERKO mice makes it difficult to further narrow down the receptor specifically implicated in the treatment effect. Use of double knockouts (ERα+GPR30KO or ERα+GPR30KO) would be necessary to more fully distinguish between these pathways.

## Authors' contributions

M.A.Y. was responsible for experimental design, data analysis and interpretation, and drafting the manuscript. Y.L. and P.C. completed animal work and ex vivo analyses. H.O. supervised the experiments and manuscript preparation. All authors have read and approved this manuscript.
